# Unilateral Multifocal Follicular Thyroid Carcinoma with Vascular Invasion and Primary Hepatic Metastasis in a Dog: First Documented Case

**DOI:** 10.3390/vetsci13010043

**Published:** 2026-01-03

**Authors:** Yoobin Kim, Hyungsan Seo, Sang-kun Jang, Sangyul Lee, Hwi-Yool Kim

**Affiliations:** Department of Veterinary Surgery, College of Veterinary Medicine, Konkuk University, 120, Neungdong-ro, Gwangjin-gu, Seoul 05029, Republic of Korea; whale8280@konkuk.ac.kr (Y.K.); shs7683@konkuk.ac.kr (H.S.); newtimming@konkuk.ac.kr (S.-k.J.); tkddbf7918@konkuk.ac.kr (S.L.)

**Keywords:** canine thyroid, thyroid tumor, multifocal thyroid carcinoma, hepatic metastasis, dog, thyroidectomy, neck mass, multiple thyroid masses

## Abstract

A 14-year-old spayed female Jindo dog presented with a firm, non-painful right-sided cervical mass. Computed tomography identified three distinct, separate masses thought to be arising from the right thyroid lobe; the largest measured 66.6 mm × 42.0 mm × 37.6 mm with an estimated volume of 56 cm^3^ and showed invasion into the right internal jugular vein. Multiple hepatic nodules were detected without evidence of pulmonary metastasis and regional lymph node involvement. Right thyroidectomy with resection of the invaded vein and partial liver lobectomy were performed. The histologic results confirmed all three masses as follicular-compact thyroid carcinomas, and the hepatic lesion as metastatic thyroid carcinoma. The dog recovered uneventfully, remained euthyroid, and showed no local recurrence over a 5-month follow-up. In human medicine, multifocality is common in papillary thyroid carcinoma and is associated with a high rate of recurrence. This report documents the first canine case of multifocal thyroid carcinoma, featuring macroscopic vascular invasion and an uncommon metastatic pattern in which the liver was affected in the absence of detectable pulmonary lesion. The presence of multifocal disease within a single canine thyroid lobe necessitates comprehensive cross-sectional imaging, meticulous surgical planning with vascular considerations, and long-term monitoring to optimize the prognosis of this carcinoma.

## Simple Summary

Thyroid cancer in dogs usually appears as a single mass on one or both sides of the neck and can sometimes be found as an ectopic form at the base of tongue or heart. In this case, a 14-year-old female Jindo dog was found to have three separate tumors growing only on the right side of the thyroid gland. The tumors were carefully removed through surgery, along with a nearby neck vein that the tumor had invaded. Some nodules in the liver were also removed and were confirmed to be the first place where the cancer had spread, even though no metastasis was detected in the lungs or regional lymph nodes. The dog recovered well after surgery and did not show any changes in thyroid hormone levels. This is the first known report describing multiple thyroid cancers on only one side of the gland in a dog. The findings from this case may help veterinarians recognize unusual forms of thyroid tumor, choose the best surgical plan, and understand how these tumors can spread in unexpected ways.

## 1. Introduction

Thyroid tumors account for approximately 1% of all neoplasms in dogs [1,2,3]. Thyroid tumors diagnosed in dogs are malignant in 90% of cases, which are carcinomas or adenocarcinomas [1,2,3]. These tumors occur most commonly in middle-aged to older and medium to large breed dogs, such as Siberian huskies, golden retrievers, and beagles [1,2,3]. Bilateral involvement has been documented in approximately 36% of thyroid tumors, and ectopic thyroid tumors at base of the tongue, ventral neck, cranial mediastinum or heart base have been reported in approximately 13% [1]. However, multifocal thyroid carcinoma has not been described in the veterinary literature.

Histologically, thyroid carcinomas are broadly classified by cellular origin into follicular and medullary types. Among follicular-origin tumors, further subtypes—including papillary, follicular, and compact variants—are recognized, with the follicular and compact forms being the most commonly reported in dogs [1,3]. Most affected animals remain euthyroid; so, many cases are clinically silent and identified incidentally. When clinical signs are present, they generally result from mass effect or tumor invasion into adjacent structures, producing signs such as dysphagia, voice change, laryngeal paralysis, Horner’s syndrome, or dyspnea [1,2,3]. Additionally, approximately 10–29% of canine thyroid tumors are functional and may produce clinical signs of hyperthyroidism, although the majority are nonfunctional [1].

Metastasis is relatively common in canine thyroid carcinoma. Up to 40–60% of affected dogs have detectable metastatic disease at the time of diagnosis [2,4]. The most frequent metastatic sites include the regional lymph nodes and lungs, followed by the liver, spleen, kidney, bone, and myocardium [1,4]. Tumor size is a significant prognostic determinant; metastatic rates approach 100% in tumors larger than 100 cm^3^, whereas smaller tumors exhibit lower metastatic potential [1,5]. Vascular invasion—either macroscopic or microscopic—is strongly associated with distant metastasis and reduced survival [1,2,4,6].

In human thyroid carcinoma, the distribution of carcinoma subtypes differs markedly from that of dogs. Whereas most of the canine thyroid carcinomas are of follicular origin, approximately 80% of human thyroid carcinomas are papillary thyroid carcinoma (PTC) [1,7,8]. PTC in humans frequently presents with multifocal and bilateral growth patterns, and multiple studies have demonstrated that multifocality is associated with poorer clinical outcomes [7,9,10,11,12]. In contrast, follicular thyroid carcinoma (FTC) represents only 5–10% of human thyroid cancers, and multifocal FTC is uncommon [9,13]. Despite the predominance of FTC in dogs, no reports exist describing multifocal FTC in veterinary medicine.

The present report describes a dog with three well-demarcated, non-continuous right-sided thyroid masses, each confirmed histopathologically as follicular–compact thyroid carcinoma. One mass exhibited gross invasion of the right internal jugular vein, consistent with preoperative computed tomography (CT) findings, and the patient demonstrated an atypical metastatic pattern, with hepatic metastasis in the absence of pulmonary involvement. To the authors’ knowledge, this represents the first documented case of unilateral multifocal thyroid carcinoma in a dog. This report details a novel manifestation of canine thyroid carcinoma, emphasizing the necessity of further research into various metastatic pattern and the clinical impact of multifocality influences on overall prognosis.

## 2. Case Description

A 14-year-old spayed female Jindo dog weighing 15.8 kg was referred to the Veterinary Teaching Hospital of Konkuk University for evaluation of a firm, non-painful right cervical mass, which had been incidentally noticed by the owner approximately two weeks before presentation. The dog was subsequently examined at a local animal hospital, where cervical radiographs, ultrasonography, and CT were performed before referral. The owner reported no signs of discomfort, dysphagia, or respiratory difficulty. The patient had previously undergone a right total mastectomy and ovariohysterectomy in early 2024, and the histology results confirmed a benign mammary gland tumor and bilateral ovarian cysts. Several months later, partial resection of the left mammary glands (glands 3rd–5th) was performed without histopathological evaluation. No recurrence of estrous or other reproductive signs had been observed since sterilization. No remarkable findings were observed on palpation of the superficial lymph nodes.

Physical examination revealed a firm, ovoid, movable mass measuring approximately 83 mm × 42 mm was palpated on the right ventrolateral neck region. The overlying skin was mildly erythematous with slight warmth, but there was no swelling or pain on palpation. Two small, firm, movable subcutaneous nodules were noted near the left second mammary gland, and each was less than 1 cm in diameter. Palpation of the superficial lymph nodes yielded no significant findings and no other remarkable findings were detected on systemic examination.

**Figure 1:**
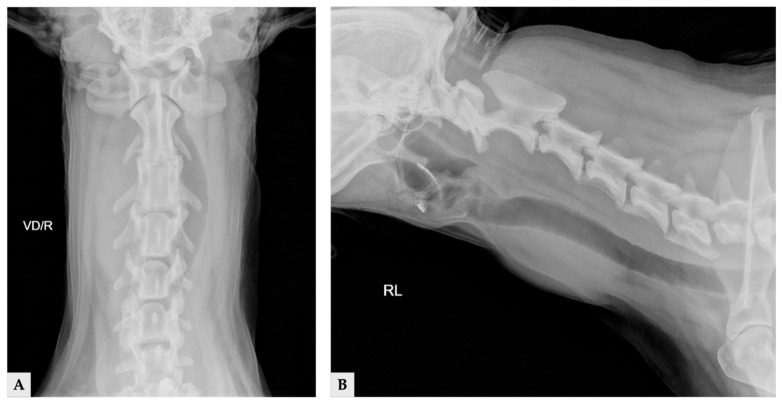
Cervical radiographic images of the day of presentation: (**A**) ventrodorsal view; (**B**) right lateral view.

**Figure 2:**
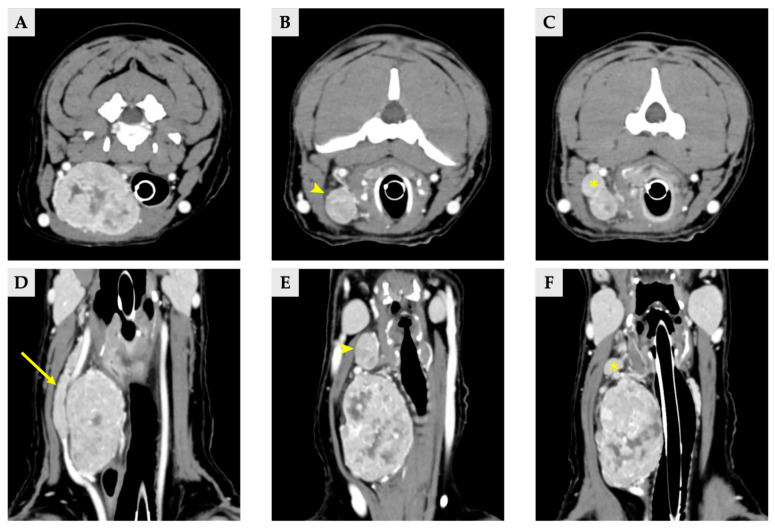
Delayed phase of CT scan image of three thyroid masses (**A**–**C**) transverse view, (**D**–**F**) dorsal view. (**A**,**D**) Images of right thyroid mass 1, demonstrating a filling effect of right internal jugular vein (arrow). (**B**,**E**) Right thyroid mass 2 (arrowhead). (**C**,**F**) Right thyroid mass 3, locating between the mass 1 and 2 (asterisk).

Radiographs demonstrated a soft-tissue opacity over the trachea at the level of C2–C5, causing leftward tracheal deviation (Figure 1). No pulmonary lesions were evident. CT confirmed three well-defined, separate right-sided cervical masses, including the largest palpable cervical mass, extending from the C1 to C5 levels (Figure 2). All three masses were clearly distinct from one another, with no visible continuity on cross-sectional imaging. Mass 1 measured 66.6 mm × 42.0 mm × 37.6 mm (approximately 56 cm^3^ in volume [14]) and originated from the right thyroid gland. It showed heterogeneous contrast enhancement and produced a filling defect within the adjacent right internal jugular vein, consistent with vascular invasion. Mass 2 measured 25.1 mm × 14.2 mm × 16.5 mm in size, located cranially to mass 1 without any structural connection, and the volume was 3.1 cm^3^ [14]. Mass 3 measured 11.5 mm × 11.5 mm × 8.6 mm in size, 0.7 cm^3^ in volume [14] and was positioned between mass 1 and mass2, lying dorsally to mass 2, and was likewise distinctly separated from the other lesions, and the total volume of three masses was approximately 60 cm^3^ [14]. The left thyroid gland appeared normal, whereas no normal thyroid tissue was identified on the right side at the corresponding anatomical level; instead, only the three masses were present. This finding supports the interpretation that these three lesions originated from the right thyroid gland.

No evidence of pulmonary metastasis was identified on CT, and the regional lymph nodes showed normal size and homogeneous contrast enhancement. However, both adrenal glands were enlarged (left: 14.1 mm cranial pole, 9.5 mm caudal pole; right: 9.3 mm cranial pole, 5.7 mm caudal pole) while retaining a normal adrenal waist configuration in ultrasonography and CT scan.

**Figure 3:**
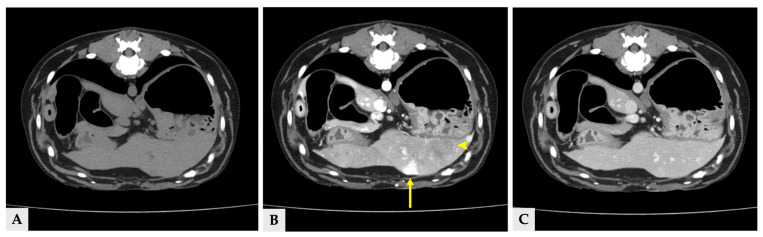
Transverse view of the CT images of several hepatic nodules at the same anatomical level. (**A**) Pre-contrast image showing a liver nodule with attenuation similar to the surrounding hepatic parenchyma. (**B**) Portal phase image at the same level demonstrating heterogeneous hyperattenuation (arrow) and hypoattenuation (arrowhead). (**C**) Delayed phase image at the corresponding level, in which the nodules show attenuation similar to the surrounding hepatic parenchyma. The yellow arrows in all three images indicate the same liver nodule (11.4 mm × 11.9 mm × 10.8 mm).

**Figure 4:**
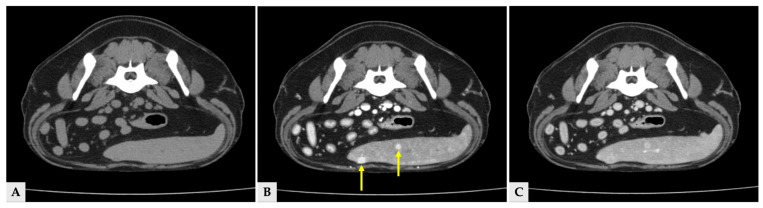
Transverse CT images of splenic nodules at the same anatomical level. (**A**) Pre-contrast image showing splenic nodules that are isoattenuated relative to the surrounding splenic parenchyma. (**B**) Portal phase image at the same level demonstrating hyperattenuated splenic nodules (arrows). (**C**) Delayed phase image at the corresponding level, in which the splenic nodules show attenuation similar to or slightly higher than the surrounding splenic parenchyma.

**Figure 5:**
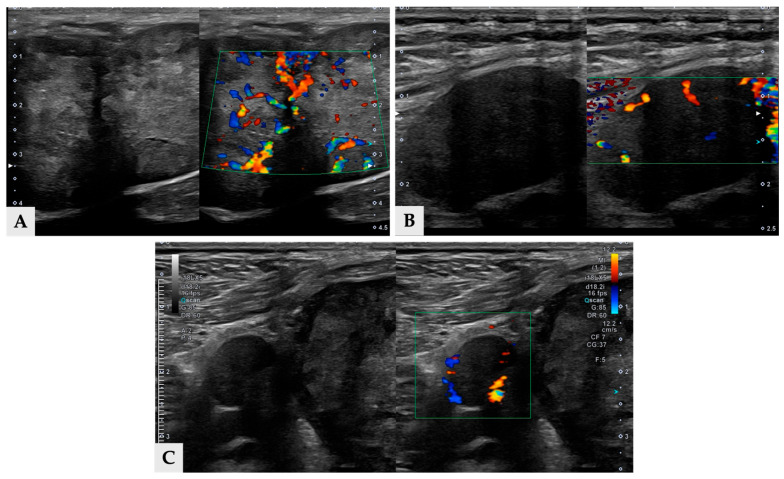
Split view ultrasonographic images of three thyroid masses (**A**) Longitudinal ultrasound image of mass 1 demonstrating heterogeneous large mass with irregular internal echotexture (**left**), color Doppler image of mass 1 showing prominent vascular signals (**right**). (**B**) Longitudinal ultrasonographic image of mass 2, which is located cranial to mass 1, revealing a well-defined, hypoechoic mass. (**C**) Longitudinal ultrasound image of mass 3 which is between 1 and mass 2, with small round, hypoechoic mass.

Multiple hyper- and hypoattenuating hepatic nodules were scattered throughout the liver parenchyma (maximum size 11.4 mm × 11.9 mm × 10.8 mm) (Figure 3), and numerous small splenic nodules exhibiting on pre-contrast and portal phase were also observed (Figure 4). Additionally, a soft-tissue mass was detected at the esophagogastric junction. Cervical ultrasonography revealed three distinct, heterogeneous right thyroid masses, with the largest mass demonstrating prominent vascularity on Doppler evaluation (Figure 5).

Blood samples were collected from the left external jugular vein. Complete blood count and serum biochemistry were unremarkable except for a mild increase in C-reactive protein. Liver enzyme activities such as alanine aminotransferase (ALT), alkaline phosphatase (ALP), aspartate aminotransferase (AST), gamma-glutamyl transferase (GGT), and total bilirubin were all within the reference range. Thyroid function tests indicated a euthyroid state, with thyroxine (T4) at 2.0 µg/dL, free T4 at 1.6 ng/dL, and canine thyroid-stimulating hormone (TSH) at 0.37 ng/mL. The cortisol level was in normal range and the urine cortisol-to-creatinine ratio (UCCR) was 13.2, ruling out hyperadrenocorticism.

Cytology test was performed under ultrasonographic guidance using a 23-gauge needle. Aspirated samples were smeared onto glass slides and stained with hematoxylin and eosin(H&E) for cytological evaluation. Cytology of mass 2 revealed changes suspicious for thyroid carcinoma. The cytology from mass 1 was non-diagnostic due to blood contamination, and mass 3 was inaccessible for sampling.

Surgery was planned to include right thyroidectomy for removal of all three thyroid masses, partial liver lobectomy of the left lateral lobe for biopsy and left unilateral partial mastectomy. The procedures were performed five days after the patient’s initial presentation.

Anesthesia was induced and maintained using standard protocols. Cefazolin Sodium (22 mg/kg, intravenous (IV); Chongkundang Cefazoline Inj. 1 g, Chongkundang, Seoul, Republic of Korea) was administered to the patient as prophylactic antibiotics, and maropitant citrate (1 mg/kg, SC; Cerenia, Zoetis, Parsippany, NJ, USA) was given as prophylactic antiemetics. The patient was premedicated with Fentanyl citrate (2 μg/kg, IV; Fentanyl Citrate Inj. (10 mL/ample(P)) Hana., Hana Pharm, Seoul, Republic of Korea) as an analgesic and midazolam (0.1 mg/kg, IV; Bukwang Midazolam Inj., Bukwang Pharm, Seoul, Republic of Korea) as a sedative. Induction of anesthesia was achieved using propofol (4 mg/kg, IV; Anepol Inc. (5 mL), Hana Pharm, Seoul, Republic of Korea), followed by maintenance with isoflurane (Terrel Solution, Kyongbo Phram, Asan, Republic of Korea) and oxygen.

**Figure 6:**
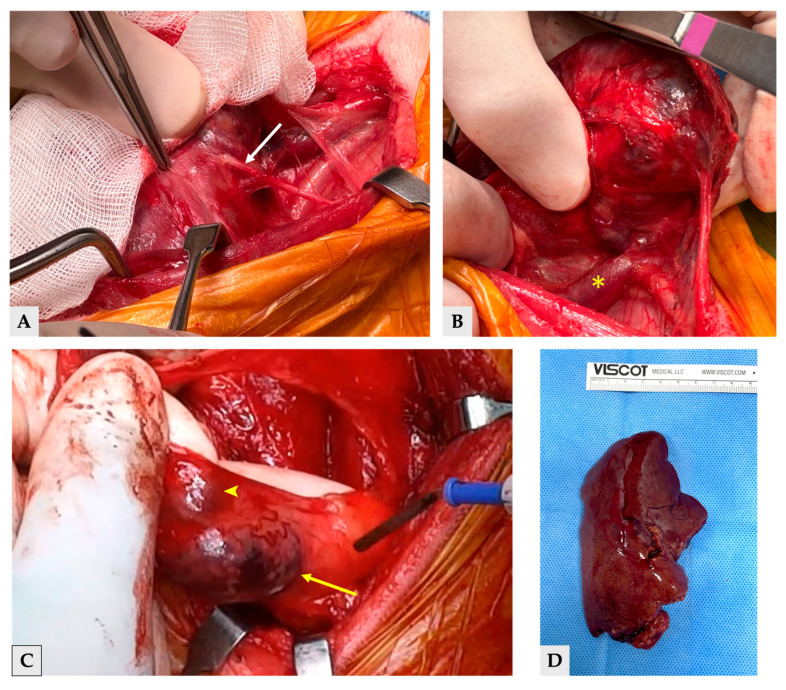
Intraoperative and gross pathologic images. (**A**) Exposure of the right cervical region. The sternohyoideus muscles have been bluntly separated, revealing the largest thyroid mass (mass 1), which is closely associated with the recurrent laryngeal nerve (white arrow) (**B**) Intraoperative view of mass 1. The image highlights the adherence of the mass to adjacent structures, notably demonstrating gross invasion into the internal jugular vein (asterisk). (**C**) Mass 2 (yellow arrow) and 3 (arrowhead) located separately within the surrounding connective tissue, providing adequate working space for removal without damage to nearby structures. (**D**) Gross appearance of the resected left lateral liver lobe, which was histologically confirmed to contain metastatic thyroid carcinoma.

Surgical preparation included clipping the patient’s hair, followed by positioning in dorsal recumbency with the neck extended over a rolled towel [15]. A skin incision was made along the palpable mass and extended proportionally to the size and orientation of the tumor to allow for optimal exposure. The sternohyoideus muscles were bluntly separated. The largest thyroid mass, mass 1 was closely associated with the common carotid artery, vagosympathetic trunk and paratracheal fascia, and it was adhered with recurrent laryngeal nerve (Figure 6A) and right internal jugular vein. Intraoperatively, mass 1 was confirmed to invade the right internal jugular vein, and an intraluminal thrombus—consistent with the preoperative CT finding of a filling defect—was identified within the vein during surgery, requiring partial venous resection while preserving the recurrent laryngeal nerve (Figure 6B). Thyroid mass 2 and 3 were located separately within the surrounding connective tissue, and because the adjacent anatomic structures were primarily displaced by mass 1, sufficient working space was available to allow for their removal without causing additional damage to surrounding structures (Figure 6C). The three thyroid masses were first surgically excised. This was immediately followed by a partial liver lobectomy of the left lateral liver lobe and a left unilateral partial mastectomy. All collected samples— including the three thyroid masses, the resected liver tissue (Figure 6D), and the mammary gland tissue—were submitted for histopathological evaluation. Each sample was collected as an excisional biopsy, immediately fixed in 10% neutral-buffered formalin and routinely processed. Histopathological assessment of all specimens was performed by a single board-certified veterinary pathologist.

**Figure 7:**
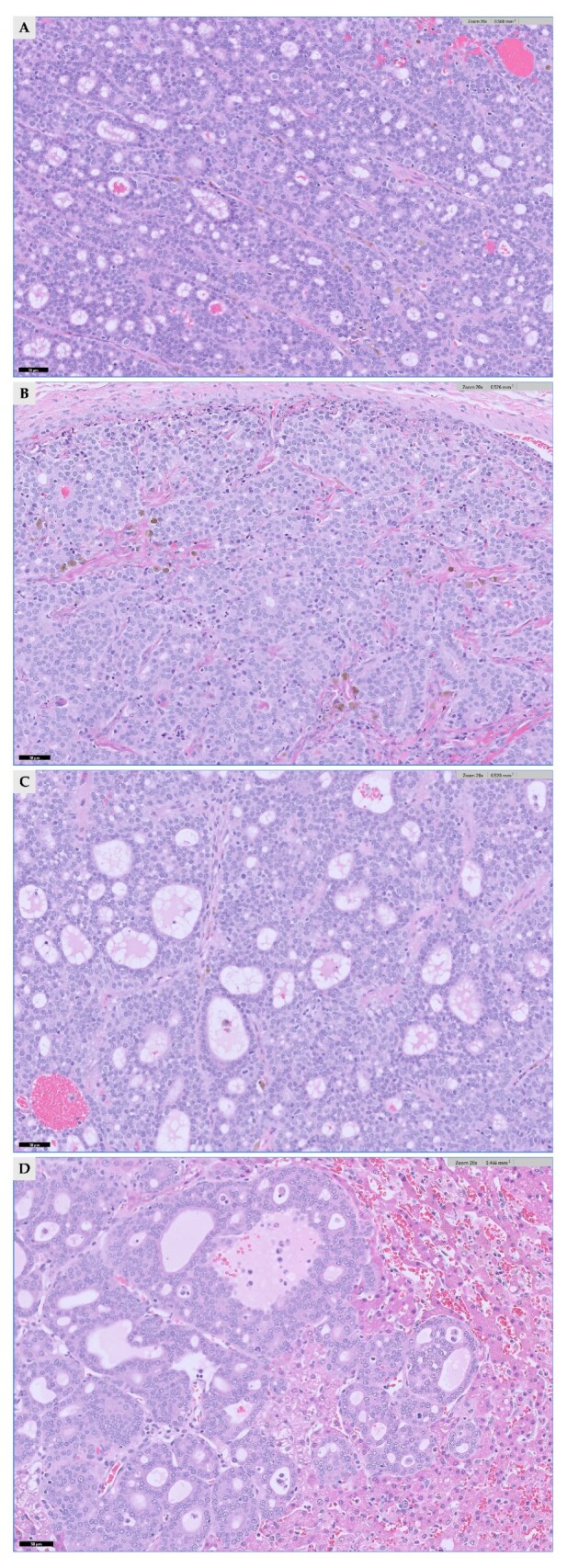
Histopathology results of three thyroid masses and left lateral liver lobe. (**A**) Thyroid mass 1: Neoplastic follicular cells arranged predominantly in follicles (H&E, ×200) (**B**) Thyroid mass 2: Neoplastic cells forming a mixture of follicles and solid packets (H&E, ×200). (**C**) Thyroid mass 3: Neoplastic cells arranged in follicles and solid packets, similar to Mass 2 (H&E, ×200) (**D**) Liver: A metastatic focus composed of neoplastic thyroid follicular cells within the hepatic parenchyma (H&E, ×200).

All three cervical masses were diagnosed as follicular-compact thyroid carcinoma by histopathology. Mass 1 was identified as a follicular-compact carcinoma with vascular invasion, whereas masses 2 and 3 were diagnosed as follicular-compact carcinomas without evidence of vascular invasion (Figure 7). The mitotic count per 2.37 mm^2^ was all different for three masses, indicating 4, 7 and 2 for mass 1, 2, and 3, respectively. The regional lymph node removed adjacent to mass 3 showed mild lymphoid hyperplasia without metastatic involvement. Histopathology of the liver confirmed metastatic thyroid carcinoma in hepatic samples (Figure 7D), along with nodular hepatocellular hyperplasia as an incidental finding. The excised left second mammary gland nodule was identified as a simple tubulopapillary cystadenoma (benign).

Postoperatively, the dog recovered uneventfully without evidence of respiratory compromise, voice change, regurgitation, or other clinical signs suggestive of recurrent laryngeal nerve dysfunction. On postoperative day (POD) 6, serum total T4 and ionized calcium remained within their respective reference ranges, and liver enzyme activities were trending downward, the dog was discharged at that time with good appetite and normal activity. By POD 14, mild seroma formation developed at both the cervical and abdominal surgical sites, which resolved within several days with conservative management, and the dog otherwise remained clinically stable. Thereafter, serial evaluations, including physical examination, serum biochemistry, thyroid hormone measurement, and thoracic radiography, revealed no evidence of local recurrence on cervical palpation and no pulmonary nodules up to approximately POD 108. Postoperative CT of the neck and thorax was not performed at the owner’s request. Follow-up was continued until approximately POD 156. During a telephone assessment at that time, the owner reported that the dog remained clinically well, and no cervical abnormalities suggestive of local recurrence were detected on owner-reported palpation. The patient is planned to undergo continued periodic evaluation at a local veterinary clinic.

## 3. Discussion

Thyroid carcinoma in dogs is generally characterized by a solitary mass arising from unilateral or bilateral thyroid gland, with reported malignancy rates exceeding 90% and a high frequency of local invasion and metastasis at the time of diagnosis [5,16,17]. Although bilateral involvement is reported in up to one-third of canine cases, and ectopic thyroid tumors occur in approximately 13% [5,18], multifocal thyroid carcinoma—particularly multiple masses from ipsilateral thyroid gland—has not been documented in the veterinary literature to date. This absence is noteworthy given that more than 90% of canine thyroid carcinomas originate from the follicular cell lineage [16,18]

In contrast, human papillary thyroid carcinoma (PTC)—which accounts for more than 90% of human thyroid malignancies—frequently exhibits multifocal and bilateral growth patterns, and multifocality is a well-recognized and extensively studied feature of human papillary thyroid carcinoma. Multifocal PTC has been repeatedly associated with more aggressive biological behavior and poorer clinical outcomes [9,19]. Although follicular thyroid carcinoma (FTC) represents only 5–10% of human thyroid carcinomas and multifocal FTC is considered rare [19,20], more than 90% of canine thyroid carcinomas originate from follicular cells [16,18], making the absence of multifocal FTC in veterinary reports particularly notable. In this context, the present case represents the first description of unilateral multifocal follicular–compact thyroid carcinoma in a dog, expanding the current understanding of the morphological spectrum of canine thyroid carcinoma.

In the present case, three anatomically separate, non-continuous masses arose from the right thyroid lobe. All three lesions shared identical histologic classification—follicular–compact carcinoma—yet lacked macroscopic or histologic continuity. Furthermore, the mitotic counts per 2.37 mm^2^ for the three masses were heterogeneous, showing values of 4, 7, and 2, respectively for the mass 1, 2, and 3, which suggests biological heterogeneity within the multiple tumor foci. Although the three carcinoma foci in this dog were anatomically separate and showed no macroscopic or histologic continuity, their shared histologic subtype and heterogeneous mitotic activity suggest the coexistence of multiple neoplastic processes within a single thyroid lobe. Despite the varied mitotic activity of three masses, the prognostic relevance of such heterogeneity remains unclear in canine thyroid carcinoma, and its interpretation is limited by the absence of established veterinary data linking mitotic variability to clinical outcome. In human oncology, papillary thyroid carcinoma (PTC) frequently presents as multifocal disease, whereas multifocality is rare in follicular thyroid carcinoma (FTC), and this difference in multifocality between PTC and FTC is hypothesized to reflect the contrasting complexity of their genetic features and oncogenic pathways [16,21]. Proposed mechanisms for the development of multifocal tumor development—largely derived from human literature—include multicentric tumorigenesis, intrathyroidal metastasis, and the involvement of embryologic remnants or segmental microvascular variations [9,13]. However, these mechanisms of multifocality have not yet been demonstrated in veterinary literature. Although the specific underlying mechanism remains unclarified, this case introduces a novel presentation of canine thyroid carcinoma not previously described in veterinary literature.

One of the most clinically relevant findings in this case was the confirmed vascular invasion within the right internal jugular vein. Vascular invasion is a well-established negative prognostic factor in canine thyroid carcinoma. In one study, it was demonstrated that both macroscopic and microscopic vascular invasion significantly reduce disease-free survival and are strongly associated with development of distant metastasis [4]. Similarly, earlier pathology studies reported that follicular-origin carcinomas frequently invade local vasculature and that vascular involvement correlates with systemic spread [22]. The present case exhibited a clear luminal thrombus visualized on preoperative CT as a filling defect and surgically confirmed during thyroidectomy. The concordance between imaging, intraoperative findings, and histopathology emphasizes the diagnostic reliability of CT in detecting vascular involvement and reinforces the prognostic implications of this feature. These findings align with current understanding that vascular invasion is a key driver of metastasis in canine thyroid carcinoma.

Interestingly, this dog exhibited an atypical metastatic pattern characterized by hepatic metastasis in the absence of pulmonary involvement. In most reported canine thyroid carcinomas, metastases are detected initially in regional lymph nodes and lungs, followed by the liver, spleen, kidneys, and bone [4,5,16]. Indeed, previous literature indicates that up to 60% of clinically detectable canine thyroid carcinomas show radiographic evidence of pulmonary metastasis at the time of diagnosis, underscoring the lung as the most common initial metastatic site [23]. In one study, all dogs with metastatic thyroid tumors were reported to have metastasis either to the lungs or to regional lymph nodes, and this study also confirmed that regional lymph nodes were the second most common metastatic site after the lungs [16,21]. Thus, hepatic metastasis without detectable pulmonary lesions and metastasis in regional lymph nodes is considered unusual. While previous reports have described hepatic metastasis in both follicular and medullary thyroid carcinoma in dogs [17], these cases uniformly involved concurrent pulmonary or extensive systemic spread. In the present case, the absence of lung involvement suggests either skip metastasis, altered hemodynamic flow, or tumor-specific biological behavior associated with the follicular compact subtype of FTC. However, none of these mechanisms has been clearly defined in veterinary medicine with respect to the metastatic behavior of thyroid carcinoma. Nonetheless, this represents one of the very few documented cases of thyroid carcinoma in dogs with hepatic-first metastasis and, to the authors’ knowledge, the only example occurring in a multifocal unilateral FTC. It is important to acknowledge that the current diagnostic imaging (Computed Tomography) may not exclude the possibility of micrometastasis in the lungs, which could represent a low tumor burden not visible radiographically and still precede the detected macroscopic liver metastasis.

Tumor size has been identified as a major prognostic determinant in canine thyroid carcinoma. Larger tumor volumes, particularly those exceeding 20 cm^3^, are strongly associated with invasive behavior and distant metastasis [4,5]. Previous studies have also shown that resectable tumors tend to be smaller (<7 cm) and freely movable on palpation [24]. In this case, the largest mass was 6.6 cm in diameter, approximately 56 cm^3^ in volume, and was movable on palpation. Although the mass was movable on palpation, its dense adhesions to the recurrent laryngeal nerve and invasion to the internal jugular vein necessitated a delicate operation for successful removal while preserving critical structures. This intraoperative outcome is consistent with reports that, despite the technical difficulty associated with invasive thyroid tumors, complete resection can be accomplished in selected cases and may significantly prolong survival [24]. The surgical complexity observed here also reflects previously described challenges in canine thyroidectomy, including hemorrhage, vascular injury, and the need for careful dissection around the carotid sheath [18].

The prognostic relevance of multifocality has been extensively investigated in human thyroid oncology, particularly in papillary thyroid carcinoma (PTC). Accumulating evidence indicates that multifocal PTC is associated with adverse clinical features, including higher rates of lymph node metastasis, advanced staging, and increased recurrence [7,25,26]. Meta-analytic data further suggest that unilateral multifocal PTC carries a significantly higher recurrence risk compared with unifocal disease [25]. This perspective has been reinforced in the 2025 American Thyroid Association (ATA) management guideline for differentiated thyroid cancer, which concludes that multifocality—especially when clinically apparent—is linked to an increased risk of recurrence. However, in humans, follicular thyroid carcinoma (FTC) represents a small proportion of differentiated thyroid cancers, and multifocal follicular thyroid carcinoma is rare, with only isolated case reports describing such presentations [27]. Consequently, the relationship between multifocality and prognosis in human FTC remains undefined due to the scarcity of available data.

In canine thyroid carcinoma, corresponding information is virtually absent because multifocal thyroid carcinoma has not been previously documented. This case therefore provides valuable comparative insight. Although three distinct ipsilateral follicular-compact masses were present, only one displayed macroscopic vascular invasion; no regional lymph node metastasis was detected, and distant spread was confined solely to the liver.

Overall, this case broadens the recognized spectrum of canine thyroid carcinoma by documenting the first known instance of unilateral multiple masses of follicular–compact carcinoma. The case also illustrates the diagnostic significance of CT in identifying multifocal lesions and vascular invasion, demonstrates the surgical challenges associated with resecting adherent or invasive cervical masses, and emphasizes the importance of considering atypical metastatic patterns in clinical evaluation. Most importantly, these findings underscore the need for larger studies to determine the biological significance of multifocality in canine FTC and to clarify whether multifocal disease should modify current prognostic frameworks or influence decisions regarding staging, treatment, and postoperative monitoring.

This report has several limitations. First, postoperative computed tomography, which had been planned for monitoring malignant recurrence or metastasis, could not be performed due to the owner’s decision. As a result, postoperative follow-up relied on thoracic radiography and abdominal ultrasonography, limiting the ability to accurately detect potential pulmonary metastasis or other visceral progression. Therefore, the absence of pulmonary metastasis or local recurrence during postoperative period in this case should be interpreted as the absence of detectable disease on thoracic radiography rather than definitive exclusion of micrometastasis. Second, in addition to the hepatic lesion that was surgically removed and confirmed as metastatic carcinoma, splenic nodules were also identified on preoperative imaging. These splenic lesions were not sampled or excised; therefore, their exact diagnosis remains undetermined. Although no enlargement of regional lymph nodes was identified on CT and the splenic nodules demonstrated imaging features more consistent with benign lesions, leading to the decision to proceed without splenectomy, the possibility of splenic metastasis cannot be definitively excluded; therefore, the term “hepatic-first metastasis” in this report is intended to indicate that hepatic involvement was the first site of distant metastasis confirmed histologically. Consequently, the overall metastatic burden and prognosis in this case may have been underestimated. Third, although bilateral adrenal enlargement was detected on imaging, endocrine evaluation (including cortisol measurement and UCCR) did not support hyperadrenocorticism, and no further diagnostic testing was pursued; thus, the underlying cause of adrenal enlargement remains unresolved. Finally, parathyroid tissue was not identified in the resected specimens. Histopathology demonstrated that none of the three cervical masses contained parathyroid tissue, and no grossly identifiable parathyroid structure was observed during surgery. Despite this, serial ionized calcium measurements before and after surgery remained within the reference interval, and no clinical signs of hypocalcemia were noted.

## 4. Conclusions

This report describes the first documented case of unilateral multifocal follicular thyroid carcinoma in a dog, characterized by three anatomically separate tumors arising within a single thyroid lobe. Despite sharing identical histologic classification, the lesions demonstrated heterogeneous mitotic activity, suggesting biological variability among the tumor foci. The presence of macroscopic vascular invasion and confirmed hepatic metastasis in the absence of pulmonary and regional lymph nodes’ involvement represents an atypical metastatic pattern compared with previously reported canine thyroid carcinomas. Surgical excision, including thyroidectomy and partial liver lobectomy, resulted in a favorable short-term outcome with stable thyroid function and no local recurrence during the follow-up period. This case highlights the significance of thorough diagnostic imaging and complete staging when evaluating thyroid masses, particularly when multifocal disease is suspected. Furthermore, the findings underline the need for additional studies to clarify the prognostic relevance of multifocality in canine follicular thyroid carcinoma and to determine whether biological behavior differs from that of solitary tumors.

## Data Availability

The original contributions presented in this study are included in the article. Further inquiries can be directed to the corresponding author.

## Abbreviations

The following abbreviations are used in this manuscript:

FTC	Follicular thyroid carcinoma
PTC	Papillary thyroid carcinoma
CT	Computed tomography
UCCR	Urine cortisol to creatinine ratio
IV	Intravenous
SC	Subcutaneous
H&E	Hematoxylin and eosin
POD	Postoperative day
ATA	American Thyroid Association
ALT	Alanine transferase
ALP	Alkaline phosphate
AST	Aspartate aminotransferase
GGT	Gamma-glutamyl transferase
T4	Thyroxine (tetraiodothyronine)
TSH	Thyroid stimulating hormone
